# Long-term daily hydrometeorological drought indices, soil moisture, and evapotranspiration for ICOS sites

**DOI:** 10.1038/s41597-023-02192-1

**Published:** 2023-05-13

**Authors:** Felix Pohl, Oldrich Rakovec, Corinna Rebmann, Anke Hildebrandt, Friedrich Boeing, Floris Hermanns, Sabine Attinger, Luis Samaniego, Rohini Kumar

**Affiliations:** 1 Helmholtz-Centre for Environmental Research, Permoserstraße 15, 04318 Leipzig, Germany; 2 Czech University of Life Sciences Prague, Faculty of Environmental Sciences, Kamýcká 129, Praha–Suchdol, 165 00 Czech Republic; 3 Friedrich Schiller University Jena, Institute of Geoscience, Burgweg 11, 07749 Jena, Germany; 4 German Centre for Integrative Biodiversity Research (iDiv) Halle-Jena-Leipzig, Puschstrasse 4, 04103 Leipzig, Germany; 5 University of Potsdam, Institute of Environmental Science and Geography, Am Neuen Palais 10, 14469 Potsdam, Germany

**Keywords:** Natural hazards, Hydrology

## Abstract

Eddy covariance sites are ideally suited for the study of extreme events on ecosystems as they allow the exchange of trace gases and energy fluxes between ecosystems and the lower atmosphere to be directly measured on a continuous basis. However, standardized definitions of hydroclimatic extremes are needed to render studies of extreme events comparable across sites. This requires longer datasets than are available from on-site measurements in order to capture the full range of climatic variability. We present a dataset of drought indices based on precipitation (Standardized Precipitation Index, SPI), atmospheric water balance (Standardized Precipitation Evapotranspiration Index, SPEI), and soil moisture (Standardized Soil Moisture Index, SSMI) for 101 ecosystem sites from the Integrated Carbon Observation System (ICOS) with daily temporal resolution from 1950 to 2021. Additionally, we provide simulated soil moisture and evapotranspiration for each site from the Mesoscale Hydrological Model (mHM). These could be utilised for gap-filling or long-term research, among other applications. We validate our data set with measurements from ICOS and discuss potential research avenues.

## Background & Summary

Europe has been hit by a series of droughts and hot spells in recent years^1^, with climatic conditions in the last decade among the most extreme in more than 250 years^2^. Quantifying the impact of these events on the terrestrial carbon sequestration capacity is crucial for understanding how future climate change may alter the terrestrial carbon cycle^3,4^. This requires direct measurements of the exchange of greenhouse gases between the lower atmosphere and the terrestrial surface performed using the eddy covariance technique^5^. Due to the high technical investment and site requirements alongside the complexity of the measurements, the application of eddy covariance is only possible at certain locations. Research infrastructure networks such as the Integrated Carbon Observation System (ICOS-RI) aim to ensure that measurements are representative and comparable through standardization and quality control^6,7^. ICOS also advances research on relevant topics. In response to the 2018 extreme compound drought and heat event in Europe, for example, the Drought-2018 initiative published an aggregated dataset of affected stations^8^, which strongly facilitated the study of the extreme event impacts^9–23^.

Despite the efforts of research infrastructure networks such as ICOS, there is still a lack of standardized datasets for quantifying drought severity. This partly arises from the lack of a uniform definition of drought severity^24,25^, preventing holistic assessment of extreme droughts and their impact^26^. For example, a meta-analysis of 564 ecological drought studies^27^ found that conditions in ~50% of all studies were within the range of normal climatic variability and therefore could not be classified as drought, and at least 30% of studies confused aridity with drought. This highlights the urgent need for uniform and standardized definitions.

To support research into drought impact on ecosystems, we provide a dataset of daily standardized drought indices tailored to each ICOS ecosystem station. We calculate three indices: the Standardized Precipitation Index (SPI)^28,29^, which is based on precipitation anomalies only, the Standardized Precipitation Evapotranspiration Index (SPEI^30^), which is based on a simplified water balance, and a Standardized Soil Moisture Index (SSMI), which is based on modelled soil moisture anomalies^31^. While a variety of other indices can be found in the literature^24,32–34^, probabilistic indices such as these allow standardized comparisons as they express the current conditions with respect to the long-term climatology of the respective location. Such standardized indices enable comparison even across larger spatial areas or between climate zones^33,35^.

While the probabilistic approach is a robust and widely used method^34^, it comes with a challenge. Robust estimation of the climatic distribution of the target variable requires 30 years of data at least^28^, and preferably as much as 50 to 80 years^36^. The first eddy covariance stations were established only in the late 1990s, with many stations being much younger. Therefore, in order to estimate the climatology, external data sources are necessary. Here, we combine long-term observational data with simulations from the mesoscale Hydrological Model (mHM)^37,38^ to provide a comprehensive drought description. Both observational and model data are compared to ICOS measurements to ensure that the data provided accurately represents the meteorological conditions at each site.

In summary, we provide a consistent long-term hydrometeorological database of drought indices that allows the comparative analysis of e.g. ecosystem responses based on standardized drought severity across different ICOS ecosystem stations. We additionally provide simulated long-term data of soil moisture and evapotranspiration, and demonstrate exemplary use-cases. We hope that the database, with its daily temporal resolution and a wide range of aggregation periods ranging from a few days to two years, will facilitate future studies on the impact of droughts on European ecosystems and ensure comparability of research results.

## Methods

### Data source and workflow

**Fig. 1:**
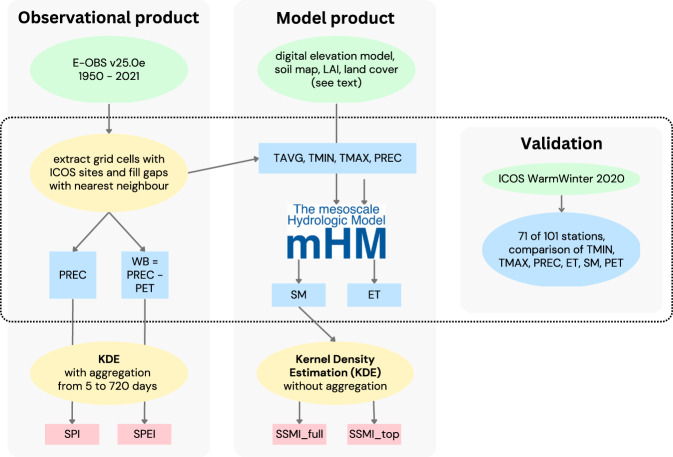
Schematic overview of the workflow. For each site, temperature and precipitation data are extracted from the E-OBS dataset and gap-filled with the nearest neighbour approach where necessary. Standardized Precipitation Index (SPI) and Standardized Precipitation Evapotranspiration Index (SPEI) are calculated using observational data for different aggregation periods ranging from 5 to 720 days. The E-OBS data are also used to run mHM along with information on elevation, soil, leaf area index, and land cover type to simulate long-term soil moisture data for the Standardized Soil Moisture Index (SSMI). All variables were compared to the respective ICOS site measurements using the WarmWinter 2020 dataset^46^.

Drought indices were calculated based on the European Climate Assessment & Dataset (ECA&D) E-OBS gridded dataset v.25.0e^39^ and soil moisture simulations realized with the mesoscale Hydrologic Model v5.11.1 (mHM)^37,38^. The workflow is presented in Fig. 1. For each ICOS ecosystem site, the respective meteorological forcing data were extracted from the corresponding E-OBS grid cell (~10 × 10 km^2^) and used to calculate indices directly (in the case of SPI and SPEI) or to run mHM to derive soil moisture simulations for SSMI. mHM was set up with the same resolution as E-OBS and the digital elevation model from USGS^40^, the soil map from SOILGRIDS^41^, the land cover from ESA^42^ and LAI climatology from NASA Global Inventory, Monitoring, and Modelling Studies (GIMMS)^43^. The underlying parameterization of the European-wide mHM setup was based on established settings, which have been evaluated in previous studies^2,44,45^.

SPI and SPEI were calculated using nonparametric kernel density estimation (KDE) with aggregation times ranging from 5 to 365 days (in steps of 5 days) and 370 to 720 days (in steps of 10 days). No aggregation was performed for the SSMI because soil moisture itself naturally reflects past meteorological conditions. The SSMI was calculated based on two different mHM soil layers, one from the upper soil layer (SSMI_top, ~30 cm), reflecting shorter meteorological fluctuations, and the other considering the entire soil layer up to 2 m soil depth (SSMI_full, ~200 cm).

**Fig. 2:**
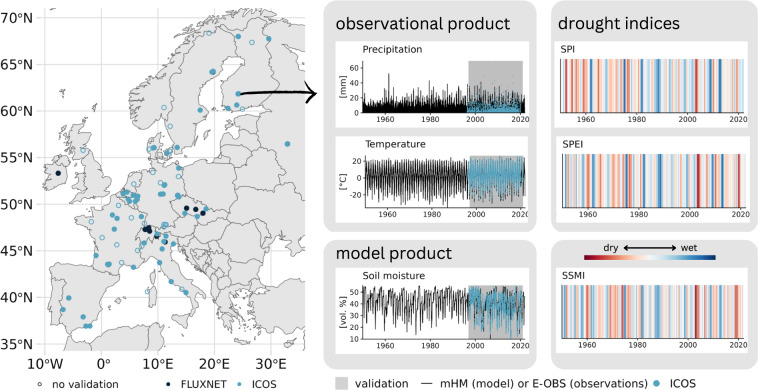
Map of ICOS ecosystem stations in Europe and data visualization for one selected station (FI-Hyy). Our dataset includes indices and simulations for each station on the map, but only the filled circles are included in the WarmWinter 2020 dataset from ICOS and were therefore used for validation. The validation dataset includes some FLUXNET stations, which are also included here. The time series for precipitation, air temperature and soil moisture shows the relative length of the validation period to the full time series we provide. Note that the validation period varies from station to station because they were established in different years.

Figure 2 illustrates the spatial coverage of our data set. Stations were selected using the ICOS station network overview (https://www.icos-cp.eu/observations/station-network) and filtering by *THEME* = “*ecosystem*”. The most recent observational data product for eddy covariance fluxes from ICOS, the Warm Winter 2020 dataset^46^ (WW20 hereafter), was used as ground truth for the technical validation. Stations included in WW20, but not in the ICOS station network, were also used (indicated by the dark blue on the map). Stations in Greenland, Israel, Congo and French Guiana were removed as E-OBS does not cover those locations. In total, we provide drought indices for 101 stations in Europe, of which 74 are validated against ICOS observational data.

### Standardized drought indices

Since its introduction in the 1990s^28,29^, the SPI has been widely applied as a robust method to compare precipitation anomalies objectively across different climate zones^47–50^, and is recommended by the World Meteorological Organization as a key drought indicator^51^. The SPEI^30^ is an extension of the SPI which additionally accounts for changes in Potential Evapotranspiration (PET), and is therefore better suited for the detection of droughts which are induced by increased air temperature^34,52,53^. In contrast to the SPI and SPEI, the SSMI is not based on observational data directly, as sufficient historical records for soil moisture do not exist. Probabilistic soil moisture indices are usually constructed from model-based soil moisture estimates^54–57^. The soil moisture index based on mHM, which is used in this study to simulate long-term soil moisture time series at the respective sites, is used in the German Drought Monitor^58^ and the South Asian Drought Monitor^59^ and has been validated and utilized for multiple applications^60,61^.

Probability-based indices are traditionally derived by fitting the variable of interest to a suitable probability density function (pdf). The corresponding standard normal deviation can be obtained from the resulting cumulative density probability (cdf) using the equiprobability transformation. The choice of the most suitable pdf is non-trivial and has been a matter of debate since the development of probability indices^29,62–64^. Instead of assuming a parametric distribution function, one can determine the density distribution from the empirical data itself^65–68^. The advantages of this approach are that no assumptions need to be made about the functional form, thus avoiding possible bias due to an inappropriate parametric model. Therefore, in this study we apply non-parametric kernel density estimation (KDE) for estimating the drought indices for all variables: P, P-PET, and SM.

We calculated daily drought indices (i.e., SPI, SPEI, and SSMI) using KDE. For a given time series of the target variable ${X}_{i,j}^{k}\left(i=1,\ldots ,n\right)$ with *j* being the time step (here, daily) and *k* the number of previous time steps used for aggregation to the current time step, the kernel density for each *j* can be obtained using a Gaussian kernel by:

1 $$\widehat{f}(x)=\frac{1}{n\cdot h}\mathop{\sum }\limits_{i=1}^{n}\frac{1}{{(2\pi )}^{1/2}}{\exp }\left[-\frac{{(x-{x}_{i})}^{2}}{2{h}^{2}}\right]$$

where *n* represents the sampling size and *h* the bandwidth. In kernel density estimation, the bandwidth selection is the highest source of uncertainty^69^. A common approach is unbiased cross-validation (CV)^31,70^:

2 $${}_{h}^{\min }\,CV(h)=\int \widehat{f}{\left(x| h\right)}^{2}dx-2\frac{1}{n}\mathop{\sum }\limits_{i=1}^{n}{\widehat{f}}_{-i}\left(x| h\right)$$

where dropping *x*_*i*_ when estimating *f*(*x*) (denoted by ${\widehat{f}}_{-i}\left(x| h\right)$) yields the least-squares CV criterion. Finally, to facilitate the comparison with classic drought categories found in the literature (e.g. in Table 2), derived based on the standard scores, we transform the KDE-based quantiles into standardized normal scores. These standardized values can be obtained from the cumulative density function F(x) by an approximation of the normal quantiles^71^:

3 $$SDI=\left\{\begin{array}{cl}-\left(W-\frac{{C}_{0}+{C}_{1}W+{C}_{2}{W}^{2}}{1+{d}_{1}W+{d}_{2}{W}^{2}+{d}_{3}{W}^{3}}\right), & {\rm{if}}\;P < =0.5,\\ +\left(W-\frac{{C}_{0}+{C}_{1}W+{C}_{2}{W}^{2}}{1+{d}_{1}W+{d}_{2}{W}^{2}+{d}_{3}{W}^{3}}\right), & {\rm{if}}\;P > 0.5,\end{array}\right.$$

where

4 $$W=\left\{\begin{array}{cc}\sqrt{-2{\rm{ln}}(P)}, & {\rm{if}}\;P < =0.5,\\ \sqrt{-2{\rm{ln}}(1-P)}, & {\rm{if}}\;P > 0.5,\end{array}\right.$$

with *P* = 1-*F*(*x*) and *C*_0_ = 2.515517, *C*_1_ = 0.802853, *C*_2_ = 0.010328, *d*_1_ = 1.432788, *d*_2_ = 0.189269, *d*_3_ = 0.001308.

Kernel estimators are known to perform poorly for bounded random variables, e.g. precipitation, in which *f*(*x*) is only supported on *R*+ = (0,+∞), as kernels can extend past such boundaries, causing leakage of probability mass^72^. To avoid erroneous estimations for *x* = 0 precipitation, we extend the non-parametric estimation for the probability of zero precipitation, similarly to the original SPI method:

5 $${\widehat{f}}_{prec}(x)=q+\left(1-q\right)\cdot \widehat{f}(x)$$

where *q* is the empirical probability of *x* = 0 precipitation.

### Estimation of potential evapotranspiration

The potential evapotranspiration required for the SPEI determination was calculated using the Hargreaves-Samani equation (H-S)^73^:

6 $$PET=0.00023\cdot RG\cdot {\left(TX-TN\right)}^{0.5}\left(TG+17.8\right)$$

where *TG*, *TX* and *TN* are the average, minimum and maximum temperature, respectively, and *RG* is the global radiation, i.e. the total of the shortwave solar radiation reaching the ground. Several methods have been proposed for estimating PET, ranging from simple empirical models with few parameters, such as the Thornthwaite model^74^, which requires only the daily or monthly average temperature, to more complex physical models, such as the Penman-Monteith (P-M) model^75^. The H-S model is a parsimonious option with low data demand and reasonable accuracy^76^, and was therefore chosen here. PET is also provided directly from E-OBS, based on the Makkink^77^ or the P-M model, but it is calculated based on an older E-OBS version and therefore covers a shorter time period than our dataset.

Global radiation is estimated based on latitude as proposed in the original SPEI publication^30^. Global radiation is also available from the E-OBS dataset, but with poorer spatial coverage and more gaps than the temperature variables. To have a consistent estimate of PET and soil moisture, we estimated global radiation as:

7 $$RG=(24\cdot 60)/\pi \cdot 0.082\cdot dr\cdot (\omega \ast {\rm{\sin }}(lat)\cdot {\rm{\sin }}(\delta )+{\rm{\cos }}(lat)\cdot {\rm{\cos }}(\delta )\cdot {\rm{\sin }}(\omega ))$$

where *dr* represents the inverse relative distance between Earth and the sun, *ω* is the sunrise hour angle, lat is the latitude, *δ* is the sun declination in radians, and *doy* is the day of the year. The solar declination can be calculated as follows:

8 $$\delta =0.409\cdot {\rm{\sin }}\left(2\pi \cdot {\rm{doy}}/365.25-1.39\right)$$

The inverse relative distance between Earth and the sun, corrected for the eccentricity of Earth’s orbit around the sun, can be calculated as follows:

9 $${\rm{dr}}=1.0+0.033\cdot {\rm{\cos }}\left(2\pi \cdot {\rm{doy}}/365.25\right)$$

and the sunrise hour angle as follows:

10 $$\omega ={\rm{\arccos }}(arg){\rm{w}}{\rm{i}}{\rm{t}}{\rm{h}}\;arg=-{\rm{\tan }}({\rm{l}}{\rm{a}}{\rm{t}})\cdot {\rm{\tan }}(\delta )$$

## Data Records

The dataset can be obtained from a Zenodo repository^78^ and comprises four files for each of the 101 sites. “[site_name]_input.csv” contains the observational data extracted from E-OBS gridded dataset v.25.0e^39^, PET estimates as well as the simulated soil water storage and actual evapotranspiration from the mesoscale Hydrologic Model v5.11.1 (mHM)^37,38^; and the remaining files contain the drought indices (“SSMI_[site_name].csv”, “SPI_[site_name].csv”, “SPEI_[site_name].csv”). Details of the variables, their units and their origin are given in Table 1. For the SPI and SPEI, the file of each site contains the estimates for various aggregation times, ranging from 5 to 720 days in steps of 5 days from 5 to 365 and steps of 10 days from 370 to 720. Each data file has a daily temporal resolution and covers the time span from 1950 to 2021.

**Table 1:** Variable description of the files contained in this data record.

Variable	Description	Source	unit
**file 1: [site_name]_input**			
Date	Gregorian calendar date	–	YYYY-MM-DD
Doy	Day of year	–	–
lon	Longtitude	ICOS	degree (°)
lat	Latitude	ICOS	degree (°)
PREC	Precipitation	E-OBS	mm
TG	Average temperature	E-OBS	°C
TN	Minimum temperature	E-OBS	°C
TX	Maximum temperature	E-OBS	°C
PET	Potential Evapotranspiration	Eq. 6	mm
RG	Global radiation	Eq. 7	Wm^−2^
AET	Actual Evapotranspiration	mHM	mm
SM_top	Soil moisture (top soil, 30 cm)	mHM	dimensionless (0–1)
SM_full	Soil moisture (full soil, 200 cm)	mHM	dimensionless (0–1)
**file 2: SSMI_[site_name]**			
Date	Gregorian calendar date	–	YYYY-MM-DD
SSMI_top	Standardized Soil Moisture Index (top soil, 30 cm)	Eqs. 1,3	standard deviation of *N*[0, 1]
SSMI_full	Standardized Soil Moisture Index (full soil, 200 cm)	Eqs. 1,3	standard deviation of *N*[0, 1]
**file 3: SPI_[site_name]**			
Date	Gregorian calendar date	–	YYYY-MM-DD
SPI_x	Standardized Precipitation Index	Eqs. 5,3	standard deviation of *N*[0, 1]
	with x = aggregation time (5,10,15...720 days)		
**file 4: SPEI_[site_name]**			
Date	Gregorian calendar date	–	YYYY-MM-DD
SPEI_x	Standardized Precipitation Evapotranspiration Index	Eqs. 1,3	standard deviation of *N*[0,1]
	with x = aggregation time (5,10,15...720 days)		

Each variable is available with a daily temporal resolution from 1950 to 2021.

## Technical Validation

**Fig. 3:**
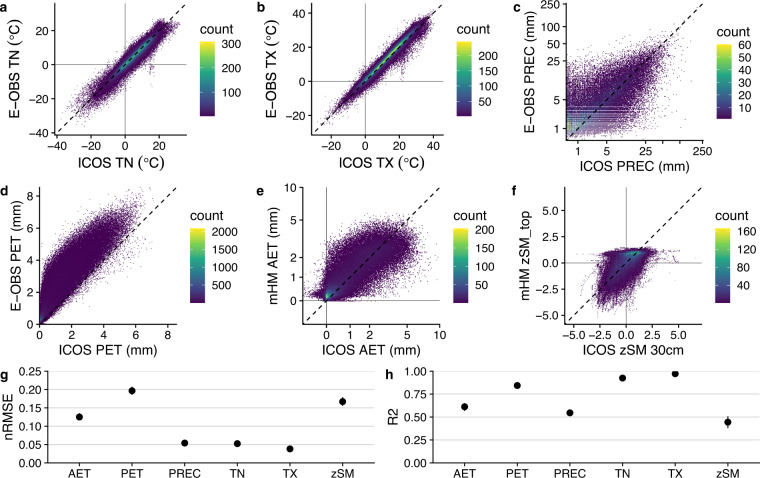
Comparison of ICOS measurements using the Warm Winter 2020 dataset and E-OBS observational data (**a**–**c**), estimated potential evapotranspiration (**d**) and simulations of actual evapotranspiration and soil moisture (**e,f**) from mHM. Note that ICOS PET is based on direct measurements of solar radiation while E-OBS PET is based on radiation estimated from latitude. Note also that soil moisture was standardized before comparison due to the difference in units between ICOS data and mHM output. Normalized RMSE and R^2^ for all variables are shown in (**g**) and (**h**).

The accuracy of drought indices depends largely on the reliability of the meteorological variables used for their calculation. The E-OBS data have been extensively used and validated^39,79^. Nevertheless, we compared the measurements at the ICOS stations with the extracted grid cells from E-OBS, with the results shown in Fig. 3a–d. The temperature variables show high agreement between E-OBS and ICOS data. Precipitation showed low error on average (in terms of normalized RMSE), but the calculated Pearson’s correlation coefficient is almost half of those obtained for the temperature variables. We find that smaller precipitation events show higher uncertainty. This is not unexpected, since precipitation is notoriously difficult to estimate at high spatial or temporal resolution^80^. However, since the indices are calculated using aggregated data from the last *x* days, this uncertainty is offset by the aggregation.

PET estimated with E-OBS data is generally higher than the observations from ICOS because the radiation is estimated from latitude only, and therefore does not include information on cloud cover. However, it has been shown that this is less relevant than the temporal consistency for SDI due to the standardization^30,81^, which is estimated by the R^2^ of 0.85. Figure 3e,f shows modelled water fluxes (namely, evapotranspiration (ET) and soil moisture) from mHM. Both modelled variables have low performance compared to the observations from ICOS. The mHM setup has a resolution of approximately 10 × 10 km^2^ and can therefore only approximately represent the conditions at specific locations. On the other hand, the soil moisture index does not require temporal aggregation, and its implications may be more straightforward because it is based directly on soil moisture information, while SPI and SPEI are only proxies for the water available to the ecosystem.

To demonstrate the information contained in the mHM output, we trained regression models for each ICOS site from the WW20 dataset using the eddy covariance fluxes from the sites as the target variable. More precisely, we trained regression models separately to predict net ecosystem exchange (NEE_VUT_MEAN), gross primary productivity (GPP_NT_VUT_MEAN), ecosystem respiration (RECO_NT_VUT_MEAN) and latent heat flux (LE_F_MDS) for the growing seasons (here May–September). In each model, we used shortwave incoming radiation (SW_IN_F) as a predictor and alternative soil moisture information as a covariate, namely soil water content measurements (from the ICOS sites, SWC_MDS_F_3) or simulated soil water content (from mHM, SM_top or SM_full). The regression models were trained using restricted cubic spline regression from the R-package “rms”^82^ with 5 knots per variable.

**Fig. 4:**
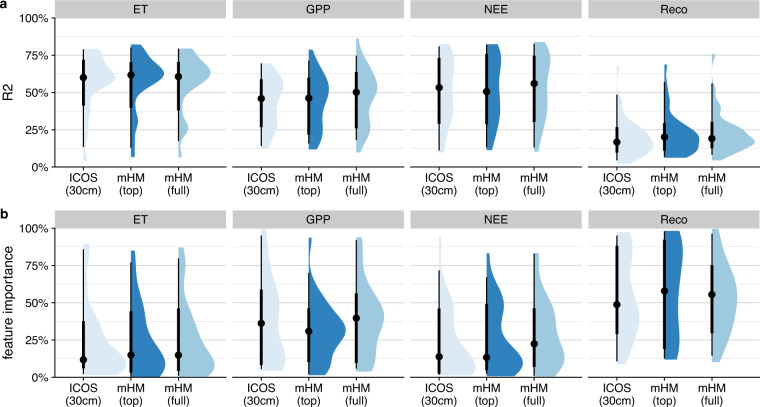
Performance of regression models for each ICOS site using eddy covariance measurements from May to September as target variable and shortwave incoming radiation as predictor, with different soil moisture products as a covariate. Panel A shows the R^2^ for each target variable and Panel B shows the corresponding feature importance based on *χ*^2^ for each soil moisture product. For mHM soil moisture, “top” refers to 0–30 cm and “full” refers to 0–200 cm.

Figure 4 illustrates that the models achieve similar performance regardless of whether the soil moisture information provided comes from observations or simulation, and regardless of whether soil moisture is of higher or lower importance to the model. We assume that the comparable performance can be attributed to a similar level of uncertainty in the datasets, despite the fact that this uncertainty arises from different causes. Uncertainty in the results from mHM is may be due to the coarse resolution representing the soil moisture conditions at the specific sites less accurately, while the observed data from ICOS contains greater noise. For a fair comparison, we only used data from days in which soil moisture measurements were available. The great advantage of the simulations, however, is that they provide gap-free time series over many years.

## Usage Notes

**Fig. 5:**
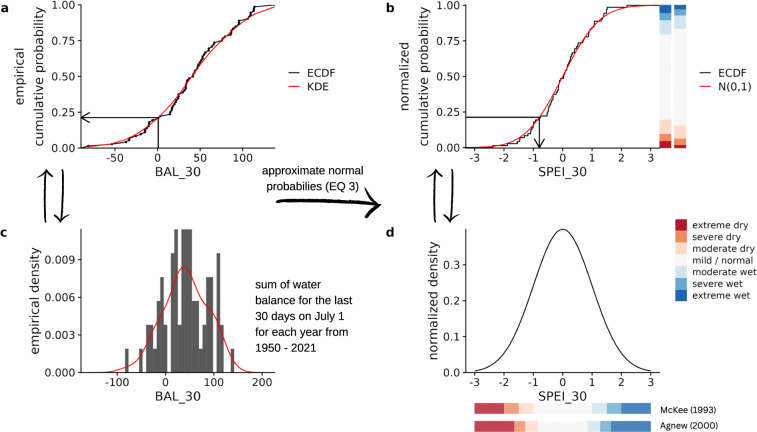
Example transformation from observational data to drought index using data from the ICOS station FI-Hyy. The sample used here is the 30-day water balance on July 1 from 1950 to 2021. Panel A shows the empirical cumulative density function (ECDF) and the estimated function using the kernel density approach (KDE). The arrow in Panel A shows the 30-day water balance on July 1, 2004, and its corresponding cumulative probability. Panel B shows how the value for the drought index (here SPEI_30) can be read for the day after the normal probabilities have been approximated. Panel C shows the histogram of the sample and the KDE, and Panel D shows the normalized density after the transformation.

In the following, we illustrate how the derivation of drought indices from climate data facilitates their utility. An exemplary transformation of observational data to a drought index is shown in Fig. 5. Here, we calculate the SPEI with an aggregation time of 30 days (as an equivalent to the SPEI of one month) for July 1 for the ICOS station “FI-Hyy”. In other words, we aim to estimate the climatic conditions on July 1 based on meteorology from the previous 30 days. To do this, we sum the water balance for the last 30 days of each year for which we obtained data (here 1950–2021). This allows us to estimate the empirical distribution function (cf. Figure 5a) of the atmospheric water balance on July 1 with Eq. (1). Figure 5c shows how the histogram of the sample relates to the estimated probability function of the KDE from Eq. (1).

In the next step, the empirical probabilities are transformed equi-probabilistically into the standard normal probabilities using Eq. (3). For example, the sum of the water balance on July 1, 2004 was 0.78 mm in FI-Hyy. The corresponding cumulative probability is highlighted in Fig. 5a. After the transformation, the probability of a given summed water balance on July 1 can be read as the standard deviation of a normal distribution as shown in Fig. 5b. A cumulative water balance of 0.78 mm corresponds to a standard deviation of −0.796, and therefore does not classify as drought (cf. Table 2). Note that this applies only to July 1. The exact value of the water deficit is less important for the calculation of the SPEI than the probability of occurrence. Rain or water deficits of equal magnitude can correspond to drought or non-drought conditions depending on the time of year of occurrence. Therefore, these steps must be repeated for each day of the year to obtain a complete time series.

**Table 2:** Proposed transformation of probabilities into drought categories.

SDI	Probability	McKee (1993)^28^	Agnew (2000)^83^	U.S. Drought Monitor (2002)^84^
<−2.00	0.023	Extreme		Exceptional (D4)
<−1.65	0.050		Extreme	Extreme (D3)
<−1.50	0.067	Severe		
<−1.28	0.100		Severe	Severe (D2)
<−1.00	0.159	Moderate		
<−0.84	0.200		Moderate	Moderate (D1)
<−0.50	0.300			Abnormally dry (D0)
<0.00	0.500	Mild	No drought	

The results of the drought index allow the definition of drought categories. Different approaches can be found in the literature, which we summarise in Table 2. McKee^28^ originally proposed a division into mild (over −1), moderate (over −1.5), severe (over −2) and extreme (below −2) drought. Agnew^83^ criticized the fact that any sub-zero SDI (e.g. below average water availability) would be classified as a drought under this definition. They suggest a classification anchored on probabilities rather than deviations. In the Agnew’s classes, moderate, severe, and extreme droughts have probabilities of 20%, 10%, and 5%, respectively, while in the original classification, they have probabilities of 15.9%, 6.7%, and 2.3%, respectively (cf. Table 2). The two different classifications are visualized in Fig. 5, both for the probabilities and the standard deviation. The U.S. Drought Monitor^84^ uses similar probability classes to those Agnew proposed, with one additional class.

**Fig. 6:**
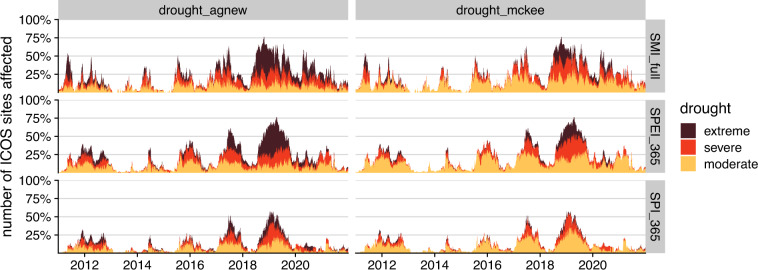
Time series of drought occurrence for the past 10 years (2011–2021) at ICOS ecosystem sites for SPI_365, SPEI_365 and SSMI_full using the two different classification approaches described.

These classification differences, although seemingly minor, can have a strong impact on drought assessment. As an example, the daily time series of the proportion of ecosystem ICOS sites affected by drought is shown in Fig. 6. We used all three drought indices with an aggregation time of 365 days for the SPI and SPEI, and applied both classification schemes described above. During the recent 2018–2020 drought and heat wave in Europe, extreme drought affected up to 40–50% of all sites during the most intense period in 2019 according to the Agnew classification, compared to only 20–25% drought according to the McKee classification. Although the number of sites affected by drought is similar, the number of sites affected by “extreme” drought is very different. This emphasizes the importance of specifying the classification scheme used to characterise drought severity levels to facilitate the comparison of research results from different studies.

**Fig. 7:**
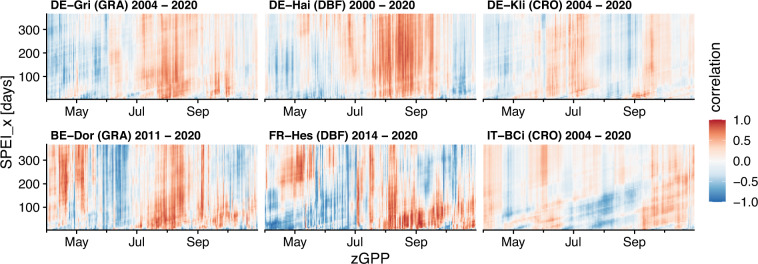
Correlation fields between daily gross primary productivity (GPP) and SPEI with different aggregation times (x = days) for six different ICOS sites. GPP was z-transformed to avoid influence from seasonality.

We anticipate that the daily temporal resolution of this dataset will enable more detailed examinations of ecosystem response to drought, as drought indices are predominantly used with monthly resolution in current research (cf. e.g. https://spei.csic.es/index.html). To get a first insight, we plotted the correlation between GPP and SPEI as a two-dimensional time series for several sites in Fig. 7. We applied a z-transformation of GPP for each day of the year to remove seasonal phenology effects, and then calculated a running Pearson’s correlation coefficient using a rolling window size of 14 days with all available aggregation levels of SPEI. Consequently, the x-axis represents the temporal development of GPP (as anomalies) during the growing season, while the y-axis represents the SPEI with increasing aggregation time, or in other words, an increasing amount of information on past conditions. As this is only an exemplary analysis of what could be done with the high-resolution dataset provided, rather than a comprehensive analysis, a more sophisticated analysis is needed to better understand how ecosystems respond to shorter and longer droughts.

In summary, we provide a long-term dataset with information on drought conditions for ICOS ecosystem sites in Europe, which additionally includes long-term simulations of soil moisture and evapotranspiration for each site. Depending on community feedback on the applicability of the data provided, we plan to update the dataset annually to provide a consistent dataset for drought research on European ecosystems. We believe that the use of the standardized drought indices provided in this dataset will improve the comparability of studies on the impacts of extreme events across the various ICOS ecosystem sites.

## Data Availability

Code for calculation of the drought indices as well as all statistical analysis within this publication is publicly available at the zenodo repository^85^. The mesoscale Hydrologic Model is an open source model and is available at https://mhm-ufz.org/.
